# From Enzymatic Dopamine Biosensors to OECT Biosensors of Dopamine

**DOI:** 10.3390/bios13080806

**Published:** 2023-08-11

**Authors:** Cristian Ravariu

**Affiliations:** 1 Biodevices and Nano-Electronics of Cell Group, Department of Electronic Devices Circuits and Architectures, Polytechnic University of Bucharest, Splaiul Independentei 313, 060042 Bucharest, Romania; cristian.ravariu@upb.ro or edusciart@gmail.com; 2 EduSciArt SRL, Iovita 2, 050686 Bucharest, Romania

**Keywords:** dopamine, biosensors, OECT, FET, aptamers

## Abstract

Neurotransmitters are an important category of substances used inside the nervous system, whose detection with biosensors has been seriously addressed in the last decades. Dopamine, a neurotransmitter from the catecholamine family, was recently discovered to have implications for cardiac arrest or muscle contractions. In addition to having many other neuro-psychiatric implications, dopamine can be detected in blood, urine, and sweat. This review highlights the importance of biosensors as influential tools for dopamine recognition. The first part of this article is related to an introduction to biosensors for neurotransmitters, with a focus on dopamine. The regular methods in their detection are expensive and require high expertise personnel. A major direction of evolution of these biosensors has expanded with the integration of active biological materials suitable for molecular recognition near electronic devices. Secondly, for dopamine in particular, the miniaturized biosensors offer excellent sensitivity and specificity and offer cheaper detection than conventional spectrometry, while their linear detection ranges from the last years fall exactly on the clinical intervals. Thirdly, the applications of novel nanomaterials and biomaterials to these biosensors are discussed. Older generations, metabolism-based or enzymatic biosensors, could not detect concentrations below the micro-molar range. But new generations of biosensors combine aptamer receptors and organic electrochemical transistors, OECTs, as transducers. They have pushed the detection limit to the pico-molar and even femto-molar ranges, which fully correspond to the usual ranges of clinical detection of human dopamine in body humors that cover 0.1 ÷ 10 nM. In addition, if ten years ago the use of natural dopamine receptors on cell membranes seemed impossible for biosensors, the actual technology allows co-integrate transistors and vesicles with natural receptors of dopamine, like G protein-coupled receptors. The technology is still complicated, but the uni-molecular detection selectivity is promising.

## 1. Introduction

Understanding the neuro-modulation processes in the human brain or animal correlates with target neurotransmitter monitoring and helps to reveal complex behaviors in neuroscience nowadays [1,2]. Dopamine (DA) is a main representative of neurotransmitters, it belongs to the catecholamine group, and it is implicated in many functions of the human brain, including emotional balance, heart control, motor control, cognition, or reward [3]. High levels of dopamine can be responsible for maniacal disorders and cardiotoxicity risks that start with hypertension and can finish with heart failure [4]. Low DA levels at different brain compartments cause several neurological diseases, such as Alzheimer’s disease, Parkinson’s disease, depression, and schizophrenia [5]. Consequently, the measurements of the DA concentrations are important in all neuro-psychiatric domains and, recently, in general medicine, too [6,7,8,9,10,11,12,13,14,15].

For in vivo DA measurements, one of the most used methods is the neurotransmitters fluorescent detection that permits the monitoring of the synaptic transmission in a few seconds response time, with a single cell precision [16]. After neurotransmitter binding, the fluorescence emission is recorded by optical transducers, like fiber photometry [16], bio-photometry [17], or characterization bio-device [18]. Specific in vivo bio-sensors can be inserted in the animal brain with implantable devices, with viral vectors, or with specific cell expression [19]. The use of the DA natural receptors would offer the best selectivity/specificity results for DA. They belong to the family of G protein-coupled receptors (GPCRs), which are expressed in the majority of living cells, including neurons and bacteria [20,21]. Very recently, the natural receptors were used to recognize elements within Field Effect Transistors (FET) using receptor-containing nanovesicles with functional GPCR, arousing interest in in vitro biosensors, too [16,22,23].

In fact, this review is rather focused on the in vitro detection of dopamine using biosensors. All known neurotransmitters were strongly explored in the last 25 years with in vitro biosensors [24,25,26,27]. The modern trends indicate changing the transducers’ size from electrochemical combinations of tens of centimeters up to sensitive transistors of a few millimeters [28,29], continuing to the down-scaling of the integrated BioFETs (Biomaterial Field Effect Transistors) and nano-devices that can include nanomaterials [30,31].

Traditional analytical methods for measuring the levels of dopamine or any other neurotransmitter include enzyme assays, capillary electrophoresis [32], high-performance liquid chromatography (HPLC) [33], mass spectroscopy [34], and electrochemical detection [28], but they suffer from high costs, expensive equipment, large size, and expensive staff training. To detect small molecules of analytes, like DA, several solid-state devices were developed. The first representative was the Ion-Sensitive Field-Effect Transistor (ISFET) [35]. Then these successively followed: electrolyte-insulator-semiconductor (EIS) [36], organic thin film transistor (OTFT) [37], Organic Electrochemical Transistor (OECT) [38], and extended-gate field-effect transistor (EGFET) [39]. For the transducer part, many solutions have arisen: one-dimensional (1-D) Si-nano-wires-FET [40], two-dimensional (2-D) nano-sheets like Graphene-FET [41], three-dimensional (3-D) nano-devices like nano-tubes [42], and conducting polymers tubes, with various advantages, such as facile functionalization [43].

However, all these bio-devices have some constraints for DA detection. Firstly, in main biological liquids, DA coexists with other electroactive species, like Uric Acid (UA) or Ascorbic Acid (AA). These acids have similar redox potential, resulting in frequent artifacts in DA monitoring by biosensors [36,44]. Additionally, the real biological samples contain other neurotransmitters and proteins besides common AA and UA interferents. Hence, additional steps in sample preparations are required. Centrifugation is necessary for blood samples, preceded by dilution. For the urine samples, only dilution before analysis is required [32]. For the cerebrally extracellular fluid sample, it is rather recommended to perform real-time in vivo analysis with miniaturized microelectrodes due to the availability of a very low quantity of fluid [32].

On the other hand, DA biosensors must change their linear range of detection from mili-molar [3,24,28] to nano-molar concentrations to obtain clinical significance [32]. The usual semiconductors used in Metal Oxide Semiconductor (MOS) transistors manufacturing offer poor subsequent catalytic properties [42,43,44,45]. To overcome these difficulties, new sensing nanomaterials (e.g., carbon nano-tubes (CNT), nano-porous layers, or meander electrodes) were proposed to be attached, deposited, or decorated onto the surface of traditional semiconductors [44,45,46,47,48,49,50].

The paper is structured in the following sections: (i) introduction; (ii) review of the main classes of DA receptors as the main engine for the biosensors evolution; and (iii) all sub-classes of organic transistors, with emphasis on OECT.

## 2. Evolution of the Dopamine Biosensors after Receptor Element

### 2.1. Enzyme-Based Biosensors

For dopamine detection, some enzymes have been exploited as receptor elements: polyphenol oxidase, tyrosinase, horseradish peroxidase (HRP), tyramine oxidase, and laccase [51]. 

The dawn of dopamine biosensors began with the immobilization of slices of plant tissue, such as green bananas, which contained polyphenol oxidase as the target enzyme. The hydrogen peroxide produced by dopamine oxidation was correlated with dopamine concentration. The DC current was measured with amperometry in a three-electrode cell, keeping pH = 7 with a phosphate buffer saline (PBS) solution. The results showed a linear current excursion of 0.1–0.8 μA per cm^2^ of the electrode for dopamine concentrations ranging between 0.4–1.6 mM [52]. Till today, the polyphenol oxidase is used as an enzymatic receptor, offering a LOD of 8 nM and a linear range of 50 nM–85 μM [53]. 

Shinohara and collaborators presented dopamine biosensors based on enzyme receptors and chemiluminescence transducers. The dopamine oxidizes under tyramine oxidase to produce hydrogen peroxide, which reacts with luminol-generating chemiluminescence, generating a limit of detection (LOD) of 10 nM and a linear range of 80 nM–500 nM [54]. 

The most popular enzyme-based dopamine biosensors used tyrosinase, connected to fluorescent or electrochemical transducers. More recently, a functionalized tungsten disulfide nano-tube was used as a dopamine work electrode for its electrochemical detection by a tyrosinase-based receptor [55]. With a linear range between 0.5–10 µM and a sensitivity of 6.2 ± 0.7 mA/M, the overall sensor performances are in agreement with similar biosensors that use carbon nano-tubes. 

Rahman designed a tyrosinase-based biosensor with multi-walled carbon nano-tubes modified with glassy carbon electrodes as work electrodes for dopamine. An important step was performed in terms of long-term stability and robust immobilization of the receptor, while interference with ascorbic acid was negligible at 1 mM dopamine. So, in 2016, the most successful sensor was produced, having a high sensitivity of 1323 mA/M^−1^ cm^−2^ and long-term promising stability, keeping 90% of its initial activity for 30 days [56].

Another enzyme that is sensitive to dopamine is laccase. Santos et al. fabricated an amperometric biosensor working with a working electrode made by carbon paste modified with laccase enzyme, which was immobilized in a glutaraldehyde agent. A linear concentration range of 0.8 μM–62 μM, with a minimal detected concentration of 0.06 μM, was accomplished, but the stability was up to 90% from a signal in only 9 days [57].

The field effect transistor-based biosensors for dopamine were extremely rare before 2015. In 2021, an Enzyme-FET with tyrosinase was fabricated for the DA measurements in fish brains in vivo [58]. A linear range of 0.1 μM–120 μM and a low detection limit of 3.3 nM were monitored. In Table 1, some features of the dopamine-sensitive enzyme-based biosensors are chronologically presented. Fluorescent techniques allowed lower detection ranges than electrochemical techniques, and an increased interest in natural receptors is noticeable, too [59,60,61].

### 2.2. Antibody-Based Biosensors

Antibodies have also become very popular receptors for biosensor development. They offer many advantages, such as high specificity, mature detection platforms, and selective binding affinity, similar to lateral flow devices or ELISA [51]. Nevertheless, antibody-based biosensors suffer from a few disadvantages, such as batch-to-batch instability, dependence on the cell’s production, and antibody production needs animal lots. We found a few papers about dopamine detection with antibody biosensors. To prepare a DA immuno-sensor, Choi et al. used fragments of monoclonal antibodies complementary to dopamine entrapped on gold nano-rods [62]. They used the localized surface plasmon resonance (LSPR) property to accomplish a variation in the refractive index around the nano-particles that is proportional to the DA concentration. The antibody preparation started with chemical reduction with 2-MEA (2-mercaptoethylamine). 2-MEA was mixed with the antibody solution to break up the Antibody molecules (Abcam) [63]. In the next process, called incubation, the 2-MEA-reduced disulfide bond central-placed within the antibody molecule assists in dissociating the thiol-functionalized fragments of the antibody. Then, the antibody-diluted solution and aqueous PBS buffer solution were inserted in a tube containing synthesized Au-Ag-Au nano-rods. The quadrupole mode peak had maximum shifting for the triblock Au-Ag-Au NR from 1088 nm to 1124 nm, superior to the peak for pure Au NR-200 nm where the variation was only from 1110 nm to 1120 nm, due to some better optical properties of silver to the refractive index changes. At that time, no information was provided about sensitivity. In 2014, Choi’s group repeated the immobilization of dopamine antibodies on gold nano-particles anchored on the surface of an electrode. They focused on the peak intensity of 575 nm with an extremely poor increase when the dopamine concentration varied from 1 nM to 1 μM [63].

Namkung et al. proposed a competitive indirect method for DA detection, immunoassay based [64]. The ELISA transducer offered the highest sensitivity to dopamine concentration, with optical absorbance of 0.91, when anti-dopamine Ab was diluted 6000 times. The method was not sensitive enough at low DA concentrations sub−0.1 μM, being efficient for the DA detection in urine.

The explanation for the lack of immuno-sensors of dopamine could be: (i) it is difficult to achieve in situ fast detection by immunoassay that becomes critical for dopamine monitoring; (ii) many interferences in the real samples are able to produce artifacts within the detection. Aptamers became better candidates for small molecule recognition, while antibodies have more difficulty for these targets.

### 2.3. Aptamer-Based Biosensors

Aptamers consist of single-stranded DNA or RNA molecules, able to bind analyte molecules with high specificity and affinity [51]. Aptamers can be synthesized for various target molecules, such as proteins or drugs, with high-temperature stability, high specificity, and selectivity. They are able to be better candidates than antibodies for molecular detection by the lock–key principle. Obviously, aptamers for dopamine were synthesized, being able to bind dopamine with improved affinity [65]. In this sub-section, only the dopamine aptasensors, without a transistor transducer, are discussed.

In 2014, Kim and Paeng composed a competitive enzyme-linked aptamer assay for dopamine detection, associating RNA and DNA aptamers [66]. The authors demonstrated that the use of the DNA homolog for RNA aptamer improves the detection limit and affinity toward DA. Explication is based on the particular nucleotide difference for DNA-aptamer and RNA-aptamer, taking into account that a lack of the hydroxyl group in the second 2’ location within the DNA molecule is correlated to its stability [66]. So, the DNA homolog was used. After a color change, a robust enzyme-linked aptamer assay (ELAA) for DA detection was applied as a transducer principle. The DA detection limit was 62 nM for the RNA-aptamer and 3.12 pM for the homolog DNA-aptamer. 

Many aptasensors are based on fluorescent transducers, being attractive for their direct measurements. These optical sensors continue to use DNA aptamer generated from the RNA-aptamer [66]. The optical transducer is based on a quenching material (e.g., nano-structured graphene oxide) that is adsorbed to a fluorophore-labeled aptamer in the dark state. When aptamer binds the dopamine molecule, the quencher is desorbed, and fluorescence is observed in the emission state, signaling the DA binding [51]. However, a very moderate fluorescence optical signal was detected with a fluorescence spectrophotometer. In 2016, Huang and collab. fabricated a DA florescent aptasensor based on Ru complex and quantum dots as fluorescence probes. The DA detection occurred with a LOD of 19 nM [67]. The method has some advantages: it is rapid, direct, low-cost, fast, repeatable, label-free, and easy-operating, and some emitted spectra can be observed by eyes, too.

Another optical aptasensor for DA detection is based on a colorimetric transducer. The successful transducer uses gold nano-particles (AuNPs) due to its strong extinction coefficient. However, colorimetric sensors provided higher LOD than fluorometric sensors. In 2018, Lin and collab. reported a DA biosensor using the artificial cerebrospinal fluid as a specimen. They fabricated a Dopamine Binding Aptamer (DBA) array that captures DA molecules from a fluid. By releasing the DA from DBA in an alkaline solution to produce DA-quinone, a color signaling was recorded: modifications from colorless to brown or associations with a strong reduction of the fluorescent signal, whether Au nano-particles were stabilized with bovine serum albumin (BSA-Au NP). The limit of detection for DA was 0.1 μg/mL if the colorimetric transducer was used or 0.5 ng/mL if the fluorometric transducer was used [68]. In 2020, Dalilirad et al. reported a biosensor of DA in human urine, where the usual concentrations are 51–478 ng/mL or 0.29–3.12 μM. The sensor used dopamine duplex DNA aptamers conjugated to Au-nanoparticles (AuNPs), 40 nm size, as a sensitive element. Hybridization between the conjugated AuNP-capture DNA and the complementary DNA in the test line generates a red stripe. Its color intensity indicated the DA concentration, with LOD of 10 ng/mL or 64.1 nM, visible with free eyes up to 50 ng/mL [69]. An innovative method combines aptasensors of DA with personal glucose meters. Upon the DA addition, the DNA-aptamer reacts with DA stimulating the aptamer hybridization, while the DNA2–invertase–AuNPs probe is forced away from the solid-state substrate. The invertase from AuNPs facilitates the sucrose hydrolysis to form glucose, detectable with a glucose meter. The method is indirect, but the equipment is a million times cheaper than a spectrometer. Glucose concentration versus the DA concentration linearly ranged from 0.08–100 μM. The DA detection limit was 0.03 μM in 2 μL which means 60 femto-molar [70].

On the other hand, dopamine, as an electroactive specie, can be oxidized with other catalysts, not necessarily enzymes. So, dopamine can be electrochemically detected with a work electrode enhanced with nanomaterials.

### 2.4. Nano-Particles Nanomaterial Enhanced Electrodes

**Figure 1:**
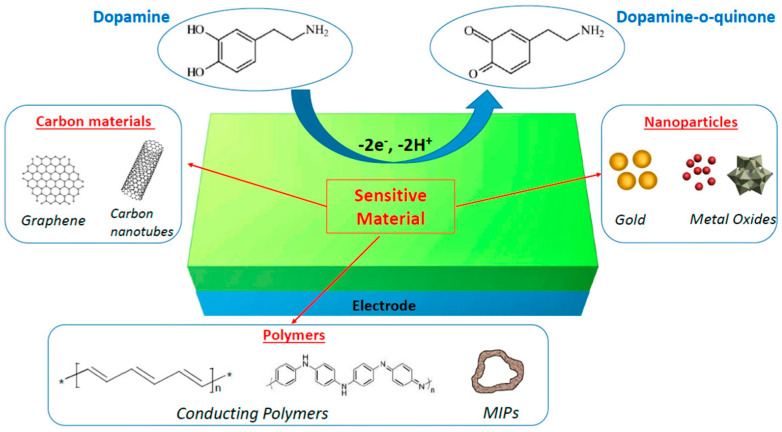
Different sensitive nanomaterial-modified electrodes for dopamine detection by oxidation (reprinted from [32] with permission from MDPI).

This sub-section reveals different oxidation agents for dopamine, such as metal, metal oxide, nanomaterials, conducting polymers, graphene, carbon nano-tubes, and molecularly imprinted polymers (MIP), also suggested in Figure 1.

Due to the huge number of studies, we resume here only a few examples to emphasize different work principles, while brief performances of other nanomaterials-based DA biosensors are included in Table 2.

In 2015, homogeneous thiophene graphene was doped with sulfur. The process was based on a solid-state reaction between sulfate and graphene oxide. The doping concentration was adjusted by the sulfate quantity. During this S-doping, some mesopores and micropores were configured on the graphene surface. Thiophene S-doped graphene presents a high electrocatalytic property for dopamine oxidation due to its high S-loading mass and multiple porous structure. Hence, the DA biosensor possessed a high sensitivity of 3.94 μM/μA, a low detection limit of 15 nM, and a signal/noise ratio of 3 [71].

In 2019, another DA biosensor used polyanilineboronic acid (PABA) functional groups as recognizing elements for dopamine. The technological process combines excellent properties of single strands of DNA functionalized on mono-walls of carbon nano-tubes (DNA-CNT) with electrochemically-derived nitrogen-doped exfoliated graphene NEG and PABA, as dopamine receptor [72]. By this method, the authors proposed a non-oxidative selective electrochemical biosensor for DA, in the presence of an important interferent, AA, in excess. This molecular anchoring fostered the electrodeposition of the nanocomposite on the work electrode, enhancing the boronic acid density as DA receptors. The developed biosensor was able to detect dopamine in a large linear spectrum, from 20 nM to 1 μM, presenting a detection limit of 14 nM [72].

On the other hand, nanomaterials combined with conducting polymers come with multiple advantages: optimized costs, biocompatibility, simple preparation, and flexible substrates. Therefore, many of them were already utilized in some applications, like, electromagnetic shielding and transparent electrodes, including DA biosensors [73,74,75]. In 2020, Park et al. fabricated a DA detector using conducting polymer nano-tubes, functionally modified with DA-aptamers. The device response was enhanced by interdigitated microelectrodes, carboxylated polypyrrole nano-tubes, pH 7.4 buffer, and a liquid-ion-gated field-effect transistor [76]. The minimum concentration of the detected dopamine was 0.1 nM. Starting with these years, working electrodes tend to be integrated into transistors more and more often. Therefore, in the following paragraph, we will refer to these types of transducers with organic transistors.

Table 2 summarizes the LOD and linear range in chronological order for DA detection with antibodies, aptamers, and nano-particles [63,76,77,78,79,80,81,82,83].

**Table 2:** The main parameters, receptor type, and transducer method, for DA detection with antibodies, aptamers, and nano-particles, in a chronological order.

Active Layer	Year	Receptor Type	Transducer Method	Range of DA Concentrations	Limit of Detection	Reference
Pd nano-particles	2008	Metal nanomaterials	Electrochemical	0.5–160 µM	200 nM	[77]
Graphene–polyaniline composite film	2012	Aptamer	Colorimetry	10–600 nM	2 nM	[78]
MWCNT	2012	Carbon nano-tubes	Electrochemical	1.2–800 µM	0.16 µM	[79]
Ru complex and quantum dots	2013	Aptamer	Fluorescence	0.5–40 μM	200 nM	[80]
Au-Ag-Au nano-rods	2014	Anti-DA Antibody	Surface plasmon resonance	1 nM–1 μM	1 nM	[63]
MoS_2_	2017	Metal oxide nanomaterials	Electrochemical	0.006–181 µM	2 nM	[81]
Au nanopillars	2018	Metal nanomaterials	Electrochemical	6–100 µM	5.83 µM	[82]
Conducting polymer/nano-tubes	2020	DA-aptamers	Interdigitated microelectrodes on liquid-ion gated-FET	1–100 nM	0.1 nM	[76]
Polypyrrole/ MoO_3_ NP	2023	Nano-particles	Electrochemical	5–100 nM	2 nM	[83]

More examples of DA biosensors containing classifications of nanomaterials are collected in another review article from 2021 [32].

### 2.5. DRD1 Natural Receptors

On the other hand, the natural receptors bound to cell membranes would be the most specific receptors offered by the living world. Their use in biosensors has been postponed for 20 years due to the technological difficulties of capturing them. But, in 2019, some authors proposed a biosensor for neurotransmitter detection, including dopamine, using their natural receptors [84]. Usually, metabotropic receptors for neurotransmitters are G-protein-coupled receptors (GPCRs). After ligand–receptor binding, an indirect action on the adjacent ionic channels happens by G-protein signaling [85]. Dopamine interacts with two kinds of GPCR receptors, usually encoded D1 and D2-like [85]. The use of these natural receptors in biosensors with optical transducers, such as fiber photometry, leads to brightness variations. In vivo dopamine biosensors were made, expressing in the animal brain some adeno-associated viral vectors. Use of false fluorescent neurotransmitters (FFN) inoculation to optically detect dopamine, reaching the accuracy of a single cell [86]. However, the existing methods are for in vivo dopamine detection and still suffer from poor temporal accuracy and require special protocols for animal inoculation (e.g., the procedure agreement for intracranial FFN infusion) [87]. Another application of drug screening needs to co-integrate GPCR receptors with electronic devices. This is a serious task for future developments of analytical devices. In 2017, a direct dopamine biosensor based on the DRD1 receptor as a D1 variant, conjugated to a CNT-FET transistor, was reported [88]. DRD1 receptor was reconstituted from its native binding pockets, offering discriminative interactions for the dopamine agonists–antagonists [89]. The monitored parameter was the equilibrium constant that characterized the affinity strength between DRD1 and the dopamine agonists–antagonists useful in drug discovery.

### 2.6. Enzyme-Free Organic Sensors 

The usual biomaterials, such as enzymes, antibodies, aptamers, or DRD1, have some disadvantages, such as complicated molecules that require further functionalization, possible post-processing inactivation of some active centers, short time activity expressed by fast expiration date for testers. On the other hand, enzyme-free organic electrochemical sensors for dopamine can reach very low LOD (in the nM range), can be used even if AA and UA are present in high concentration, and they are cheap, small, and flexible [90].

In 2021, Patella and collaborators reported a robust electrochemical DA biosensor, replacing the biomaterials with an active electrode made by Au or Pt nano-particles linked to reduced graphene oxide [90]. Here, the organic material was used to confer a flexible substrate. The technology was facilitated by the electrodeposition of the next layers, quantified by parameters like precursor concentration, deposition time, and potential. The best parameters for DA detection were conferred by the Pt nano-particles: a linear range of 100–1000 nM, maximum sensitivity of 7.19 nA/nM, and a low limit of detection of 62 nM [90].

On the other hand, metal–organic framework (MOF) materials seem to present promising characteristics for DA detection, plus a further immunity to other interferents than AA and UA, such as sulfuric acid, glucose, sodium hydroxide, and potassium chloride. Generally speaking, MOF consists of a crystalline microporous material containing a metal ion and an organic linker. In 2022, Moallem et al. produced a simple electrochemical DA biosensor that uses Cu as metal ions and 1,3,5-benzene tricarboxylic acid (BTC) as an organic linker [91]. But in 2023, Rajaitha et al. reported a lanthanum-based electrode, able to electrochemical detect dopamine. The optimal active electrodes contain La-BTC/CNT, being produced by the hydrothermal method. The DA detection reached a LOD of 73 nM and sensitivity expressed in current density as 2.96 nA/(nM cm^2^) [92].

Sometimes, the spontaneous or potential-induced polymerization of dopamine to Polydopamine (PDA) can occur. PDA is an adherent polymer that can be separately deposited on a wide diversity of materials to construct biosensing platforms [93,94].

For instance, Qu et al. reported a DA biosensor, with almost zero interferents, in terms of AA and UA, due to the acid phosphatase (ACP) used as a polymerization agent for DA [93]. Essentially, the PDA has been grown on a covalent organic framework (COF) surface, having ACT as a catalyst. Hence, the DA detection was feasible in a linear range of 0.5–50 μM with a detection limit of 160 nM [93]. Recently, a PDA functionalized graphene-based biosensor (PDA-GR) successfully electrochemically detects dopamine in a linear range of 7–297 μM, with a detection limit of 1μM [94]. This hybrid PDA-GR nanomaterial becomes a promising material for DA detection, promoting water solubility, diminishing the GR reunification, and keeping the produced coating suitable both for electroless metallization and functional organic adherence layers.

## 3. Dopamine Biosensors with Organic Transistors

Organic electronic devices were developed many years ago, starting from OLED applications in displays [95], followed by integrated electronic devices on flexible substrates [96], organic solar cells [97], biocompatible devices [98], biomimetic devices [99], and more applications of these transistors in the field of biosensors [100,101].

Mostly, there are three types of Organic Thin Film Transistors (OTFT) as the main exponents of the organic transducers for biosensors: organic field-effect transistors (OFETs), electrolyte-gated organic field-effect transistors (EGOFETs), and organic electrochemical transistors (OECTs) that will be presented and compared in this section in terms of the technological strategies and bioelectronics performance for the dopamine detection. Compared to biosensors with inorganic transducers, their OTFT-based counterparts possess larger surface areas achieved through simpler technology and room temperature for organic material processing, allowing the co-integration of biosensors on flexible substrates.

In addition, the transistors must work with liquid media for biosensing. These often cause corrosion or other chemical attacks that disrupt and sometimes irreversibly damage the transistor. That is why OFETs, compared to inorganic FETs, present an additional advantage when their construction itself includes liquid electrolytes, which allows them to operate in aqueous solutions in different configurations.

Nevertheless, the selectivity and sensitivity of the OFET-based biosensors still demand supplementary enhancement. One direction is to improve the electronic properties of the organic semiconductor, either by new doping methods, such as the incorporation of Fe^+3^ ions [101], or by using organic dopants such as para-amino-benzoic acid [102], or by alternating solvent solutions [101,102,103]. It was demonstrated that a low quantity of poor solvent like acetone, added in a larger amount of good solvent like chloroform for polymers, increases threefold the mobility and transconductance of OFET [104]. A second direction envisages the spatial arrangement of the constructive elements of the OTFT and the sensitive elements of the biosensor, generating those three classes of organic transistors, which will be further addressed in turn.

### 3.1. Dopamine Biosensors with OFET

**Figure 2:**
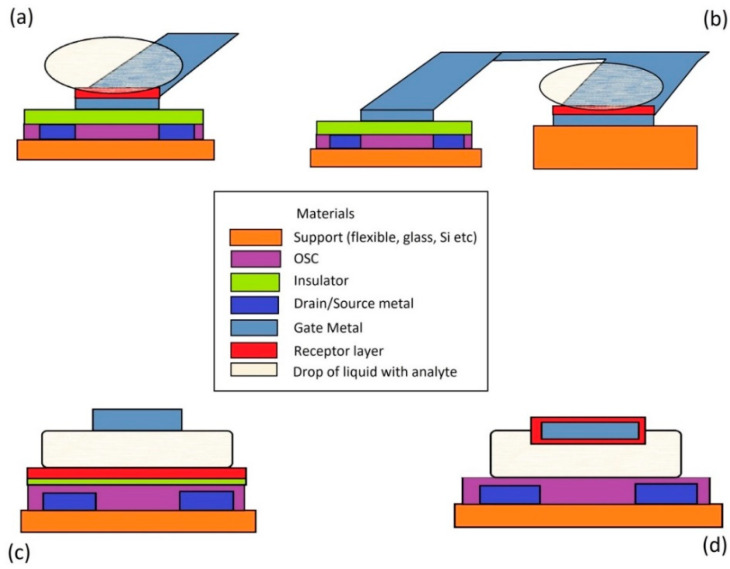
The configuration of (**a**) OFET, (**b**) OFET with extended gate, (**c**) EGOFET, (**d**) OECT.

OFETs are mainly based on the same principle as any FET, including MOS-FET, except the semiconductor is organic instead an inorganic one. The general principle is also fulfilled for OFET: the channel charge density is controlled by gate biasing. The difference consists in the type of channel. A cross-section through an OFET in Top Gate Bottom Contacts configuration is available in Figure 2a. OFET works with an accumulation channel (e.g., composed of holes in a *p*-type semiconductor and activated by a negative gate voltage) versus the MOS-FET works with an inversion channel. In the OFET biosensor, in addition to a constant external gate biasing, the variations of the electric charge on the gate are proportional to the analyte concentrations in order to produce deviations of the drain-source current and threshold voltage of the transistor. Frequently, the FET part is separated from the receptor membrane that is deposited over the gate insulator or over the substrate as an Extended Gate OFET transistor, Figure 2b.

For instance, a biosensor able to detect C-reactive protein (CRP) used an OFET transducer, determining a LOD under 1 μg/mL [105]. Here, the working principle was of an immuno-sensor reduced to an OFET biosensor. This was so that the immobilized CRP antibodies in the transistor gate change the drain-source current and the threshold voltage of the device when they bind to the CRP antigen [105].

For dopamine detection, another OFET with an extended gate was recently proposed. The active film was indium-zinc oxide (InZn_x_O_y_). It was deposited by sol-gel technology on an organic substrate made from PET [106]. The extended gate configuration uses functionalization for a synthetic receptor (4-carboxyl-phenyl-boronic acid) and is able to covalently bind the DA molecules on the target surface. The biosensor sensitivity was 10.69 mV/ decade of dopamine concentration, with a fine linearity of 0.998. The linear detection range was 1 fM − 1 nM, and the limit of detection was 0.5 fM for this DA biosensor [107].

In some usual diseases, DA concentration levels are higher than in a healthy state if the measurements are made in urine samples, reaching values up to 2.5 μM [108]. An OFET with an extended-gate electrode covered by a DA receptor always represents a solution. An additional start advantage is the disposable, extended detector part being efficient from the hygiene perspective and waste recycling. Using laccase as an enzymatic receptor, the OFET became much more sensitive to dopamine than to adrenaline and noradrenaline [109].

So, in 2023, Ohshiro et al. fabricated an enzymatic OFET biosensor, with a laccase mediator for DA oxidation, from urine samples [110]. The recognition principle was based on dopamine oxidation by laccase with N-ethylphenazonium moiety as a co-mediator. For the transistor, the insulator was AlO_x_, the organic semiconductor was PBTTT-C14 polymer deposited by drop-casting, and the drain and source were Au electrodes of 30 nm thickness. The configured channel had a 50 μm width and 100 μm length. An extended-gate gold electrode with a thickness of 100 nm was created on PEN film and functionalized with a SAM-based mediator, able to bind the enzyme. The laccase receptor was immobilized by glutaraldehyde by the usual cross-linking procedure [29]. OFET was biased under 3 V for gate and drain-source voltages. The measurements for different DA concentrations between 1 μM and 500 μM showed a threshold voltage variation from 0 to −0.12 V and a time response of 70 s. The estimated limit of detection of 0.19 μM indicates the application of this OFET-based biosensor for DA determination in human urine.

### 3.2. Dopamine Biosensors with EGOFET

Unlike the above OFET devices, the EGOFET has an electrolyte solution as a separation layer between Organic Semiconductor (OSC) layer and gate electrode, Figure 2c. In this way, two electric double layers are developed—one at the electrolyte/gate interface and another at the electrolyte/receptor–insulator–OSC interface. The existence of an insulator is not compulsory. Consequently, both the organic semiconductor and gate electrode can be used as sensing areas for target molecules.

Casalini et al. reported the first EGOFET-based dopamine biosensor based on SAM technology to immobilize the receptor on the gate electrode. The SAM receptor was integrated by 4-formylphenyl boronic acid and cysteamine, and it was able to directly bind the target neurotransmitter. As a result, the dopamine adsorption near each interface changes the capacitance of each double layer, transmitting specific signals for selective dopamine detection [111]. All the DA testers were buffered with PBS solution at pH 8.5, offering a detection limit down to the pico-molar scale. The calibration curve was the dependence of the threshold voltage (Vth) shift versus the DA concentration. After the calibration curve inspection, this biosensor indicated a Vth deviation of 50 mV for A DA concentration of 10^−12^ M and a Vth deviation of 350 mV for a DA concentration of 10^−3^ M.

### 3.3. Dopamine Biosensors with OECT

A next representative is the organic electrochemical transistor (OECT) that was introduced by White et al. in 1984 [112], with outstanding performances in biodetection due to a low cost of fabrication, simple processes, intrinsically amplifying characteristics, biocompatibility, and efficient operation in electrolyte solutions, as an ion-electron converter [113]. OECT lets the drain and source metallization in contact with the accumulation channel of the organic semiconductor, while the gate metal rests in contact with the electrolyte from the tested liquid [101]. Here, an initial drain current still exists in the absence of any gate biasing under a non-null drain-source voltage due to the conduction through the neutral channel. At a positive gate voltage, the positive ions are rejected by the gate, and they inject holes from the electrolyte into the channel. In this way, the channel receives much more positive electrical charge carriers than usual, changing its conductivity and also its drain-source current. If the gate voltage is recovered to zero, then the injected electric charge from the semiconductor is transferred to the electrolyte, and the drain-source current comes back to the initial value.

Compared to the OFETs and EGOFETs-based biosensors, OECTs deliver higher transconductance due to a stronger electronic–ionic interaction, keeping in touch the ions in electrolyte with the electrical carriers within OSC during the doping and dedoping processes [49]. Furthermore, an OECTs-based biosensor works at lower operational voltages, uses simple device structures, and refers to real-time and label-free measurements, offering robust biological detection.

The usual material for OECT is polythiophene-doped polystyrene sulfonate (PEDOT: PSS) as a semiconductor, which is deposited by spin-coating or inkjet printing processes. The extra mixing with poly(ethylene glycol) (PEG) or poly(vinyl alcohol) (PVA) of the biofunctionalized PEDOT: PSS is frequently used for biosensing purposes [101]. Then, graphite, Au, or Pt are the usual materials for the gate electrode [48]. A recent dopamine OECT biosensor was created in this way, offering the best sensitivity for Pt gate electrode, biased at 0.6 V, with a dopamine LOD sub-5nM, making it a favorable candidate for DA detection in human blood [49].

The liquid-ion-gated organic field-effect transistor is one of the most advanced OECT representatives. Park et al. reported in 2020 such a kind of DA biosensor that combines conducting polymer nano-tubes to entrap the receptor element consisting of DA-specific aptamers, interdigitated microelectrodes, and carboxylated polypyrrole nano-tubes (CPNT) as organic nanomaterials [76]. A family of the output characteristics of the CPNT aptasensor was measured, for 120 nm nano-tube diameters, when the gate voltage ranged from 0 to −100 mV and the source-drain voltage increased from 0 V to 100 mV. This CPNT aptasensor allows clinical tests for patients with DA deviations specific to schizophrenia or Parkinson’s disease. In agreement with these DA intervals, the dopamine biosensor followed a linear range of 0.6 to 6.25 ng/mL or 3.9 to 40.4 nM [76]. The detection limit was 0.1 nM.

Aptamers with a phosphate backbone were produced by the systematic evolution of ligands by exponential enrichment (SELEX) [114]. Consequently, the sensitivity and selectivity of a DA biosensor were tested on real DA samples released from PC12 cells [23]. The DA binding pocket is constituted of five nucleotides (C-G-T-G-T and G-C-A-C-A) that interact with the hydroxyl groups [115,116] from position three in the DA molecule. The opposite electrical charges were induced in the transistor body as the CPNT layer, by the phosphate backbone of aptamer, before and after DA binding. It is obvious that the induced charge in CPNT doubled for each captured DA molecule, enhancing the sensitivity [116].

Another exponent of the OECT group is that they serve wearable biosensors based on nanomaterials and textile fibers. This kind of OECT fiber-based transistor was developed in 2019 to detect dopamine as a wearable biosensor [117]. The main materials were PVA-*co*-PE nanofibers and polypyrrole nanofiber networks [107]. Both fibers stand for the source-drain electrode and the gate electrode, which were distanced at 3 mm by the gel electrolyte immobilization. The sensitivity of the DA biosensor with organic electrodes was better than the sensitivity of the device with gold and platinum electrodes. Additionally, the biosensor offered long-term stability, with a linear range of detection from 1 nM to 1 μM and excellent selectivity for interferents like UA, AA, and glucose [107]. As a general observation, the transistor size is micronic: length of 900 μm, width of 200 μm, and thickness of the active layer of 38 μm. Therefore, the detection limit cannot reach pico-molar ranges.

Recently, Liang et al. combined an organic electrochemical transistor with a flexible polyimide substrate to facilitate the immobilization of DA aptamers. The gate electrode was performed by Cr/Au/Cr metallic layers, while OSC was Poly(3,4-ethylenedioxythiophene) doped with polystyrenesulfonate (PEDOT:PSS).

**Figure 3:**
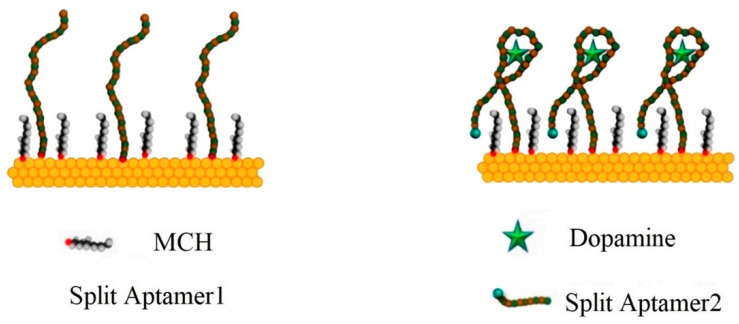
The initial receptor structure with anchored Aptamer1 strand on gold electrode (left side), and the assembling in the Aptamer1/Dopamine/Aptamer2 sandwich, after DA binding by a second split blue-labeled Aptamer2 (Reproduced from [117] with MDPI permission).

A half fragment of the receptor (aptamer1) is covalently bound to Au-electrode, while the second fragment (aptamer2) is attached for DA highlighting [117], using methylene blue label at the redox group at the distal tail, Figure 3.

Firstly, the Au-electrode surface was incubated through 0.5 µM aptamer1 that binds to the self-assembling monolayer, using thiol–Au bonding. In the next stage, the aptamer-modified electrode was sunk in 2 M NaCl aq. solution to release the aptamer2 and analyte molecules.

**Figure 4:**
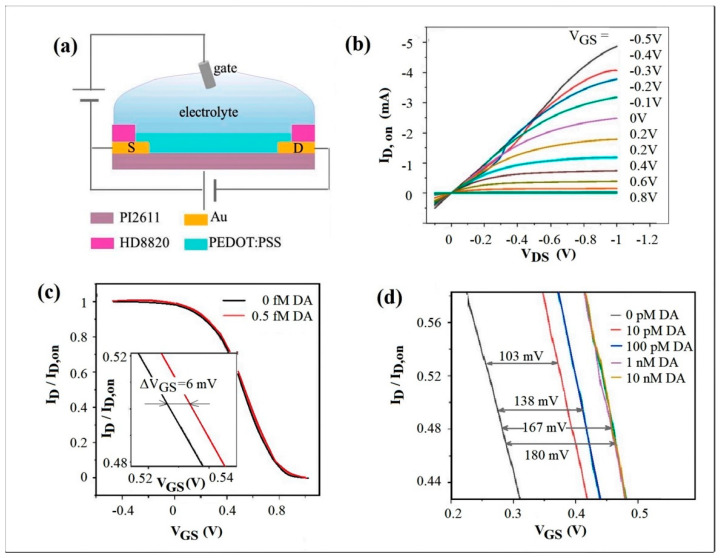
(**a**) The functional OECT structure; (**b**) the output static characteristics measured in the “on” state at different gate voltages; (**c**) the transfer static characteristics measured at V_DS_ = −50 mV in absence of DA in the tested solution and at a minimum concentration defining LOD of 0.5 fM; (**d**) deviations of the transfer characteristics with different shift voltage, proportional to the DA concentration ranging from 10 pM to 10 nM (Reproduced from [117] with MDPI permission).

As soon as the dopamine target molecules were dispersed, they fostered the coupling of the two separate aptamer fragments. The association creates multiple aptamer1/dopamine/aptamer2 sandwich structures that increase the redox concentration at the electrode surface, facilitating the charge transfer. This is so that the OECT-based biosensor exhibits an ultralow detection limit under 0.5 fM, which is one of the lowest reported LOD for DA biosensors [117]. The schema of OECT is available in Figure 4a, and the output characteristics are presented in Figure 4b.

A concentration of 0.5 fM produced an offset in the transfer characteristic of the OECT of approximately 6 mV (Figure 4c), demonstrating this ultra-low limit of DA detection. 

Figure 4d presents different shifts in the transfer characteristics toward higher gate potentials: 103 mV, 138 mV, 167 mV, and 180 mV that were measured for dopamine concentrations of 10 pM, 100 pM, 1 nM, and 10 nM, respectively. For a DA concentration higher than 1 nM, the transfer curve shifts showed a weak dependence on the DA concentration. The optimal linear range of detection was obtained between 5 fM and 1 nM.

Previously, Liao et al. reported an OECT dopamine biosensor with two types of gate electrodes: graphene-modified gate electrodes and chitosan-modified Pt gate electrodes. In both cases, the detection limit cannot be decreased under 5 nM [118].

Tang et al. also achieved a study of the OECT sensitivity to DA, using PEDOT:PSS organic semiconductor with diverse materials as gate electrodes: graphite, Au, and Pt. Pt offered the lowest limit of detection of 5 nM [38,49].

Another OECT that is able to measure dopamine in artificial sweat is based on (PEDOT:PSS) semiconductors combined with textile fibers [119]. This OECT was a very low power consumption device because its operation voltages were sub 1 V, and it consumed a power less than 0.1 mW [120]. For this device, the DA linear range was 1–100 μM, while the limit of detection was 50 nM [119].

**Figure 5:**
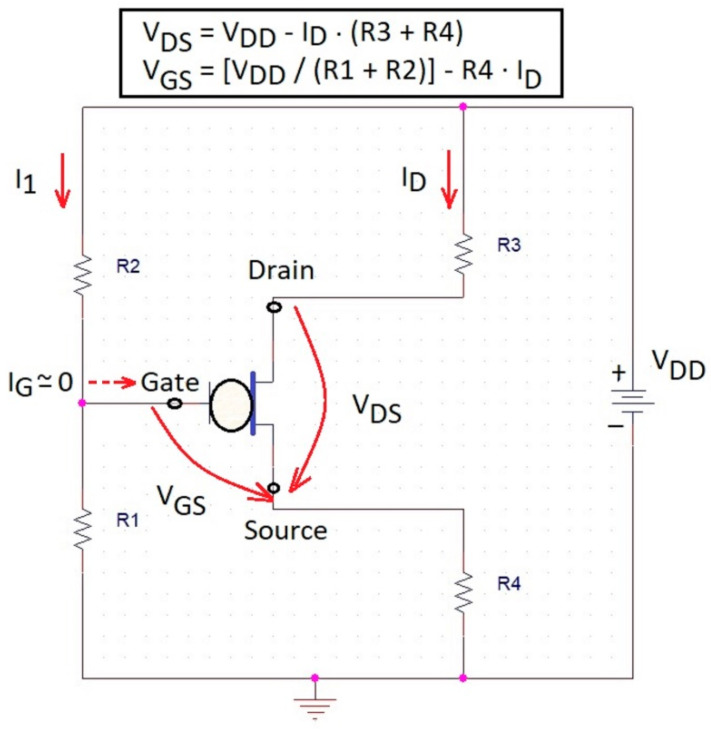
The OECT symbol and a biasing network with a sole V_DD_ source for an OECT transistor.

Moreover, OECT does not need either three electrodes measurement, nor does it need reference electrodes or counter electrodes to build these devices besides their readout devices. Nevertheless, the measurement of OECT transistors requires the application of two voltages and the measurement of two currents. Fortunately, there are solutions to simultaneously ensure convenient V_DS_ and V_GS_ drop voltages, by a unique power supply, V_DD_, and some resistors, as in the example from Figure 5. An electronic symbol is proposed for OECT among terminals—Gate, Source, and Drain in Figure 5. On the other hand, the existence of two currents in the OECT will always bring complications to the measurement scheme. Obviously, future optimizations will have to keep the drain current (I_D_) as firm as possible and the gate current (I_G_) as low as possible to stimulate the amplification property. In this case, the voltages, V_GS_ and V_DS_, can be calculated with the formulas at the top of Figure 5, considering I_G_ << I_D_ or I_G_ negligible. The co-integration of a reference electrode near OECT would be a backup solution. However, there are solutions that are known to reduce the gate leakage current by isolation, as in FETs [102], by changing the gate metal shape [120], or by modeling the transistor gate current as in the case of a triode [121].

If years ago, the use of natural receptors seemed more like a Sci-Fi story, increasingly, in recent years, natural receptors could be integrated together with organic OECT transistors. In the previous sections, we already noted these trends [36,122], but in this paragraph, an OECT-based biosensor is briefly described for the selective recognition of dopamine. OECT uses human dopamine receptor (hDRD1) conjugated to a conducting polymer membrane made by multidimensional carboxylated poly(3,4- ethylenedioxythiophene) (MCPEDOT) nanofibers, with nano-rods as the semiconductor layer. The natural receptors, hDRD1, belong to the GPCR family, and they were expressed in E-coli bacteria. They were immobilized onto the MCPEDOT surface, with grace to its amine groups that bound to the carboxyl groups of the MCPEDOT membrane. The hDRD1—MCPEDOT-based OECT presents the fastest time response, less than 2 s, high dopamine sensitivity, and a limit of detection of approximately 100 fM [122]. However, the technology is still complicated, expensive, and requires multiple facilities. The technology is a complex joint of organic semiconductor technologies, nanomaterials synthesis, bio-technology of DR natural receptors expression within E-Coli bacteria, cloning technology for the hDRD1 gene by polymerase chain reaction, specific processes of DR binding to the nanomaterials, drain-source electrodes made by Cr 20 nm on Au 200 nm deposited by thermal evaporation followed by a lift-off process, microfluidic channels configuration by Micro or Nano-Electro-Mechanical Systems (MEMS or NEMS) technology, SEM/TEM confirmations, and fluorescent labeling technology for dopamine receptors, including the receptor molecular weight confirmation by Western blot analysis using antibody technology on mouse trials.

### 3.4. Some Future Directions

New bio-devices inspired by micro-nano-electronic technologies were developed for single-cell or single-protein analysis. They combine transistor schemes of nanometric size with microgrippers, micropipettes, or microneedles—simply to be co-integrated by MEMS or NEMS techniques [123]. The use of gold-gate nanopores, FET, has been proved to permit a single molecule recognition by nano-porous material in the gate area of a field effect transistor [124].

Moreover, a nano-sized needle-type OECT was recently constructed by a single/double barrel of carbon nanoelectrodes functionalized with a conducting polymer to detect dopamine in a wide concentration range: from pico- to micro-molar range. The device works under an operation bias point V_DS_  = −0.3 V and V_GS_ = −0.9 V to exhibit a linear response of 0.002–7 μM and a limit of detection of 1 pM [125].

Another direction of OECT development envisages biosignal amplification using transistor properties. Tybrandt et al. used a PEDOT:PSS OECT to amplify the fast scan cyclic voltammetry (FSCV) signal. The sensing electrode was constructed by a 10 μm gold square plate, offering a channel length of 20 μm and a channel thickness of 60 nm. Biasing this OECT transistor to V_DS_ = −0.3 V successfully amplified the FSCV signal, in unshielded conditions, for DA concentrations in the sub-micro-molar range [126].

Recent research proposed a neuro-device [127] that opens the doors toward artificial synapses but is simultaneously sensitive to the DA concentration. This is a real development in biomimetic devices [99], serving the goals of neuromorphic electronics [96]. In this discussed case, the device is operated as a DA biosensor, reaching ultra-low detection limits, sub 1 pM [127]. The transistor body consists of PEDOT:PSS organic semiconductor, patterned on polydimethylsiloxane, as a flexible substrate. The squared electrical pulses received on the input terminal propagate through the transistor body were recorded by the output terminal in a similar manner as the pre- and post-synaptic stimulus. The transfer curve of the device depends on the dopamine concentration and stimulus frequency, mimicking the facilitating or depressing responses [99,127].

## 4. Conclusions

At the dawn of biosensors, the first dopamine detectors used stand-alone enzymes, or enzymes from plant tissues, to assist in the oxidation of dopamine. The electrochemical sensors that were used in the past offered linear detection ranges of the order of 1 mM–100 mM and detection limits of up to 1 mM. The replacement of the working electrodes from the electrochemical combined with transistors represented an important step in the miniaturization of dopamine biosensors. In this way, the detection limits were pushed below nano-molar values. All these premises led to the inclusion of organic transistors, such as OFET or OECT, inside the biosensors, including those for dopamine. The inclusion of the newest types of receptors, such as DNA-related aptamers, or natural receptors, such as hDRD1, make some direct DA biosensors from these transistors. High-sensitive and selective measurements for dopamine, with a linear detection range between 0.1 nM–10 nM and frequent LOD of 50 fM, make these biosensors suitable for all types of clinical samples collected from humans: blood, urine, or saliva. Future directions seem to abandon Immuno-FET for dopamine, reduce the Enzyme-FET for DA detection, and lean more on OECT-based aptasensors.

Dopamine wearable biosensors tend not to be a negligible sector, combining textile materials made of polymers with configurations of organic transistors and nanomaterials activated by last-generation receptors, which have proven equally promising detection ranges and excellent time response of 1 s. Advantages, like ultra-low power consumption (<1 mW power, <1 V operation voltage), could be the next key points toward an ecological economy, which will include the biosensors sector, too, considering that the actual nano-transistors are getting closer to fulfilling these conditions.

Despite the expansion of different types of biosensors and the widest range of receptors and transducers, including OECT transducers, there are still no dopamine biosensors on the market. Possible causes would be too much diversity of biosensors and materials distracts the attention of industry and marketing companies, lack of standardization of biosensors on the market, lack of cohesion between transistor manufacturers and biosensor manufacturers, and possible economic and industrial ambitions. Other times, the rapid expiration of the testers (e.g., less than 6–9 months) or their simple exhaustion from markets often leads the client to the electronic device without finding matching testers. The solution must be a stable and standard technology at a low price, which means a large series production, extending the expiration time, and ideally creating recyclables or multi-use testers. Since the testers are inserted into electronic readers, it is desirable that the transducers be transistors, too. The extraordinary success of electronics nowadays consists not of a huge number of transistors, despite hundreds of known transistors. Today, in industry, only two types are series produced: bipolar and MOS-FET transistors. This is why they benefited from ultra-optimized technology and an ultra-large scale of integration that ensured invincible prices. In 2023 the use of an ultra-large pallet of nano-bio-materials coincided with the moment of reaching sub-20 nm dimensions for transistors. A preliminary solution could be conducted by EGOFETs, where only the part with the extended gate on which the receptor is integrated should be removable and re-renewable. All these goals can be touched only by a real interdisciplinary collaboration in the future and stability in technology.

## Figures and Tables

**Table 1:** Comparisons between different DA biosensors, enzyme-based.

Type	Year	Enzyme	Transducer Method	Range of DA Concentrations	Details	Reference
Banana tissue biosensor	1990	Polyphenol oxidase	Electrochemical: amperometry	0.4–1.6 mM	3-electrodes in PBS	[52]
DA detector from PC12 cell	2007	Tyramine oxidase	Luminescence	1 nM–1 mM	In sample PC12 cells	[59]
Direct DA Biosensor	2012	Laccase	Voltammetry and electrochemical impedance spectroscopy	0.5–13 mM	Gold–Agaricus bisporus laccase electrode	[60]
Fluorescent biosensor	2015	Tyrosinase	Fluorescence of C_3_N_4_-TYR	0.01–1000 mM	In human urine sample	[61]
MW-CNT- Biosensor	2016	Tyrosinase	Amperometric biosensor	50–1000 µM	MWCNT on Glassy carbon electrodes	[56]
Tungsten disulfide nano-tubes Biosensor	2019	Tyrosinase	Chronoamperometric response	0.5–20 µM	WS2-COOH nano-tubes on glassy carbon electrodes	[55]
Colorimetric sensor	2020	Tyramine oxidase	chemiluminescence	80 nM–500 nM	Hydrogen peroxide reacts with luminol	[54]
FET-Biosensor	2021	Tyrosinase	In vivo monitoring	sub-1 μM–120 μM	Regenerated field effect transistor	[58]
Amperometric biosensor	2022	Laccase	Cyclic voltammetry	0.8 mM–62 mM	Au-nano-particles carbon paste electrode	[57]
Graphene biosensor	2022	Polyphenol oxidase	Electrochemical: cyclic voltammetry	50 nM–85 μM	PEDOT–GO	[53]

## Data Availability

Not applicable.
